# Cervical and Thoracic Spine Mobility in Rotator Cuff Related Shoulder Pain: A Comparative Analysis with Asymptomatic Controls

**DOI:** 10.3390/jfmk9030128

**Published:** 2024-07-24

**Authors:** Daniel Manoso-Hernando, Javier Bailón-Cerezo, Ignacio Elizagaray-García, Pablo Achútegui-García-Matres, Guillermo Suárez-Díez, Alfonso Gil-Martínez

**Affiliations:** 1 CranioSPain Research Group, Centro Superior de Estudios Universitarios La Salle, Universidad Autónoma de Madrid, 28023 Madrid, Spain; daniel.m@lasallecampus.es (D.M.-H.); javier.bailon@lasallecampus.es (J.B.-C.); or alfonso.gil@salud.org (A.G.-M.); 2 Unidad de Fisioterapia, Hospital Universitario La Paz-Carlos III (IdiPAZ), 28029 Madrid, Spain; pabloachutegui@gmail.com (P.A.-G.-M.); gsdiez@yahoo.es (G.S.-D.)

**Keywords:** shoulder pain, RCRSP, physical examination, cervical spine, thoracic spine, ROM

## Abstract

Rotator cuff related shoulder pain (RCRSP) is a prevalent clinical presentation characterized by substantial diagnostic uncertainty. Some of this uncertainty relates to the involvement of the cervical and thoracic spine as a source of or contributing factor to RCRSP. Thirty-two RCRSP cases and thirty-two asymptomatic controls (AC), recruited from Hospital La Paz-Carlos III between March 2023 and September 2023, were matched for age, gender and hand dominance. Assessed variables included cervical, thoracic range of motion (ROM) and neck disability index (NDI). Independent *t*-tests were used to compare each of these measurements and multiple linear regression was used to examine the capacity of neck or psychosocial variables to predict the variability of the NDI. The RCRSP group had significantly reduced cervical rotation [RCRSP (111.14 ± 22.98); AC (130.23 ± 21.20), d = 0.86, *p* < 0.01] and flexo-extension ROM [RCRSP (112.47 ± 2.07); AC (128.5 ± 17.85), d = 0.80, *p* < 0.01] as well as thoracic spine flexion [RCRSP (33.02 ± 1.14); AC (34.14 ± 1.01), d = 1.04, *p* < 0.01], extension [RCRSP (28.63 ± 0.89); AC (27.37 ± 0.89), d = −1.40, *p* < 0.01], right rotation [RCRSP (40.53 ± 10.39); AC (54.45 ± 9.75), d = 1.38, *p* < 0.01], left rotation [RCRSP (39.00 ± 11.26); AC (54.10 ± 10.51), d = 1.39, *p* < 0.01] and a significantly increased NDI score [RCRSP (17.56 ± 7.25); AC (2.47 ± 3.25), d = −2.69, *p* < 0.01]. The variables best explaining neck disability were central sensitization index and SF-12 total score (adjusted *R*^2^ = 0.75; *p* < 0.01). These results suggest that clinicians should assess cervical and thoracic spine mobility in patients with RCRSP.

## 1. Introduction

Shoulder pain ranks among the most prevalent musculoskeletal conditions, with an estimated occurrence ranging from 7 to 26% [1]. Rotator cuff related shoulder pain (RCRSP) is considered the most common subgroup of shoulder pain [2]. RCRSP is an umbrella term that encompasses a spectrum of shoulder conditions, including subacromial pain syndrome, subacromial impingement syndrome, rotator cuff tendinopathy, bursitis, and symptomatic partial and full thickness rotator cuff tears [3]. It was proposed to avoid uncertainties associated with scientifically outdated diagnoses, to help the patient make sense of their experience of shoulder pain and weakness [4], and to actively move away from the term subacromial impingement syndrome [5,6].

The paramount objective in the professional practice of physical therapist is to ascertain a functional diagnosis [7]. However, RCRSP etiology is not clear [8,9,10,11] and physical examination based on special orthopedic tests to identify the source of shoulder pain has been challenged [12,13,14,15,16,17,18,19].

A further component of this dilemma is the role of the cervical spine as a source or contributing factor to RCRSP. Research investigating the association between neck and shoulder pain has found a robust association between the presence of rotator cuff lesions, upper limb pain and disability and cervical spine pain [20,21,22,23]. Furthermore, it becomes manifest that a subset of individuals presenting with RCRSP harbors a pertinent cervical component devoid of overt radiculopathy (i.e., cervical kinematics and postures demonstrably impacting shoulder discomfort and kinesthesia) [24,25,26]. Zhang et al. also demonstrated that degenerative pathology within the cervical spine may predispose a patient to rotator cuff tears [23]. On the other hand, the existence of upper-limb disorders and muscular weakness is associated with neck problems, potentially influencing the progression or management of these conditions [27] as it was also described by Gumina et al., where shoulder injuries can alter trapezius kinematics resulted in neck pain [28].

Moreover, the contributory role of the thoracic spine in shoulder biomechanics has been previously investigated [29,30,31,32,33,34]. Different studies found a relationship between thoracic spine position and reduced shoulder range of motion (ROM) in healthy population [29,30] whereas another study has demonstrated that approximately 15° and 9° of thoracic extension mobility is required for full bilateral and unilateral shoulder flexion [31]. However, recent systematic reviews have shown that thoracic kyphosis may not be a major contributor to the development of shoulder pain [32,34]. Due to this disagreement in the literature, the assessment of thoracic ROM instead of thoracic posture may be more relevant in this population, as proposed in the literature [33,34].

It is widely acknowledged that in the clinic, the cervical and thoracic spine should be examined and ruled out before a more specific diagnosis involving the shoulder is made [33,35] but the optimal methods for doing this remain unclear [36]. To the authors’ knowledge, this is the first study that assessed both the cervical and thoracic spine ROM in RCRSP patients. Therefore, the aim of this study was to establish the differences in the cervical and thoracic spine ROM in patients with RCRSP and asymptomatic subjects.

## 2. Materials and Methods

A single blinded observational cross-sectional study was conducted at Carlos III University Hospital in Madrid.

### 2.1. Participants

Ethical approval for this study was granted by Hospital Universitario La Paz-Carlos III Human Ethics Committee (approval: 2022.505, HULP code: PI-5428) in October 2022. Written informed consent was obtained from each participant. Participants were recruited from Hospital La Paz-Carlos III waiting list between March 2023 and September 2023. Asymptomatic participants were recruited through advertisements. The reporting of this study conforms to the Strengthening the Reporting of Observational Studies in Epidemiology (STROBE) statement [37].

### 2.2. Inclusion and Exclusion Criteria

Adults with persistent RCRSP (rotator cuff tendinitis or tendinosis, subacromial impingement syndrome, partial rotator cuff tear, and atraumatic rotator cuff tear) were diagnosed by a Consultant Orthopedic Surgeon, following a physical examination and confirmed by ultrasound scan or magnetic resonance imaging (MRI), that persisted longer than the normal tissue healing process timeframe.

To be included in the control group, healthy adults matched by age, sex, and dominant arm with the RCRSP subjects group were recruited with no history of shoulder, neck, or upper back injuries and no reports of painful symptoms in any of these areas in the previous 12 months.

Both case and control group subjects were excluded if participants had a diagnosis of frozen shoulder, severe shoulder arthritis, cervical radiculopathy, acromio-clavicular joint dysfunction, or shoulder instability. Likewise, patients were excluded if glenohumeral instability was identified by a grade 2 or 3 anterior, posterior, or inferior load and shift test (assessed objectively), had history of shoulder dislocation, or history of trauma which required surgery. Equally, if they were on a waiting list for shoulder surgery or previously had shoulder surgery, had gone under physiotherapy treatment, or had a shoulder corticoid injection in the last 6 months, they were excluded. Patients with a diagnose of systemic or neurological disease (type II diabetes was not screened for), recent (within previous two years) or current pregnancy, dementia or psychiatric disorders that could potentially interfere with the study participation and comprehension, or had taken analgesic medication or NSAIDs (Non-steroidal anti-inflammatory drugs) 48 h prior the physical examination were also excluded.

### 2.3. Procedures

Preceding the initiation of the evaluation, participants were informed about the physical examination protocol and questionnaires to fill in. The shoulder pain and disability index (SPADI) [38] was employed to further describe the RCRSP group, while the neck and disability index (NDI) [39], the 12-Item short form survey (SF-12), assessing the impact of health on an individual´s everyday life [40], and the Central Sensitization Inventory (CSI) designed to identify patients who have symptoms that may be related to central sensitization or central sensitivity syndromes [41] were measured in both groups. Body chart and the visual analogic scale (VAS) for pain were used to obtain pain measurements when at rest and at movement [42]. Physical examination was conducted by an experienced musculoskeletal physiotherapist with over a decade of clinical experience.

The sequential testing order for all participants was as follows:

#### 2.3.1. Cervical Active ROM (AROM)

Cervical AROM was measured by the Cervical Range of Motion (CROM; Cervical Range of Motion Instrument, Performance Attainment Associates, 1988, University of Minnesota) device, a clinical tool commonly used to measure cervical ROM [43]. Numerous studies have reported strong validity and reliability of the CROM device affirming the clinical value of the instrument. It was shown to have moderate to good intra-rater and inter-rater reliability of 0.84 to 0.96 and 0.73 to 0.94, respectively, for cervical ROM [44,45,46].

Cervical AROM in flexion and extension, right/left lateral flexion, right/left rotation, and neutral position, protraction/retraction were measured for each subject. Participants were instructed to perform three consecutive repetitions of each movement to calculate the mean value. Data were recorded in degrees of movement. To minimize measurement variability due to differences in body and head position, consistent procedures were implemented to establish a standardized body posture and a neutral head and neck position at the start of each motion measurement. During both practice and subsequent measurement trials, participants were seated in a straight-back, wooden-frame chair with an upright posture, ensuring the low and mid-back regions were in contact with the backrest. Feet were positioned flat on the floor, and upper extremities were placed at the sides with shoulders relaxed. The CROM placement followed the technique described by Reese and Bandy [47] with procedural steps adapted [46,48,49]. Two sets of eight measurements were performed. If a subject did not correctly follow the tester’s instructions during any cervical movement, the measurement was not taken, instructions were reiterated, and the movement was repeated.

#### 2.3.2. Thoracic AROM

Thoracic spine flexion and extension in the sagittal plane were measured using the Ott’s sign [50], which have shown adequate intra- and inter-rater repeatability and reproducibility [51]. The body positioning for the practice and subsequent measurement trials included having each subject sit in a straight-back, feet flat on the floor, and upper extremities positioned at the sides with the shoulders relaxed. For measurement, the most prominent spinous process of the cervical spine (C7) was detected and marked in the relaxed sitting subject. The landmark 30 cm caudal to C7 is equally marked. Changes in the length when the patient bends maximally forward and maximally backward are measured with a tape. Lengthening of 2–4 cm and shortening of 1 cm are normal values [50].

The seated rotation test, as described by Johnson et al. [52], was applied to measure thoracic rotation AROM. The participant was seated with hips and knees flexed to 90°, and a 21-cm diameter ball was positioned between the knees to restrict lower limb movement during thoracic rotation. To standardize upper extremity position, a 105-cm-long, 2.5-cm-diameter polyvinyl chloride pipe marked at its midpoint was utilized. The pipe was placed across the chest, with the participant’s arms crossed over it. A goniometer was aligned parallel to the ground at the midpoint between the T1 and T2 spinous processes. The stationary arm of the goniometer was oriented away from the direction of rotation, remaining parallel to the initial position. Measurements were taken three times on both the right and left sides. This test has shown good intertester reliability (ICC = 0.87; 95% Confidence Interval (CI) = 0.77–0.93) and excellent intratester reliability (ICC = 0.91; 95% CI = 0.84–0.95) [53].

### 2.4. Sample Size

The sample size was calculated based on data from a previous pilot study (*n* = 15) using the G Power 3.1 (University of Düsseldorf, Düsseldorf, Germany) program [54]. The required sample size to detect differences between groups in cervical lateral flexion ROM was 64 subjects in total, with an effect size of 0.63, α error of 0.05, and statistical power of 0.8 (1–β error). Considering the thoracic spine, the sample size required to detect differences between groups in thoracic extension ROM was 54 subjects, with an effect size of 0.68, α of 0.05, and power of 0.8 [55]. Therefore, 32 subjects per group were included in the study.

### 2.5. Data Analysis

Data were analyzed using IBM SPSS Statistics Version 22. Before conducting the *t*-test, the assumption of normal distribution was tested with the Shapiro–Wilk test. Descriptive statistics (mean, standard deviation, range, standard error) were calculated for each physical assessment variable.

Related to the principal goal of the study, cervical and thoracic ROM comparisons between groups were analyzed using independent Student *t*-tests or chi-squared tests for quantitative or qualitative variables, respectively. The level of significance was set at 95%.

In regard to the secondary goal, aiming to establish the variables best explaining neck disability, Pearson’s correlation tests (r) were first performed to analyze correlation between variables. Correlation coefficient below 0.3 indicated weak correlation, between 0.3 and 0.69 indicated moderate correlation, and above 0.7 a strong correlation [56]. According to correlation test results, only the three independent variables with highest values of (r) Pearson´s were entered into a predictive model [57,58].

Then, multiple backward linear regression models were used to examine the capacity of neck or psychosocial variables to predict the variability of the neck disability index (NDI). The strength of the association was determined using coefficient (B), *R*^2^, adjusted *R*^2^, and *p*-values. Standardized beta coefficients (b) were included for all predictors in the final model to allow direct comparisons between different predictor variables. The level of the significance was set at *p* < 0.05.

## 3. Results

Thirty-two RCRSP patients and thirty-two age, gender, and dominant arm matched control subjects met our inclusion criteria. In RCRSP cases, onset of pain symptoms was 39 months (±20.04 months). Participants with RCRSP had significantly higher body mass index (BMI) than those without shoulder symptoms (*p* = 0.04) (Table 1).

The results for the cervical and thoracic examination comparison are expressed in Table 2. Cervical AROM was significantly smaller in the RCRSP group compared to the asymptomatic group for cervical flexo-extension and total rotation (*p* < 0.01). No significant differences were found in cervical total lateroflexion (*p* = 0.05), cervical neutral position (*p* = 0.75), protraction (*p* = 0.39), and retraction movements (*p* = 0.39). The NDI was significantly higher in the RCRSP cases compared to the matched control subjects (*p* < 0.01). There was significantly less thoracic AROM for all movements (*p* < 0.01) in the RCRSP subjects than the matched asymptomatic group (Table 2).

The correlation model for the variables included is presented in Table 3. There was a moderate inverse correlation between NDI score and both cervical flexo-extension and total rotation (r = −0.407, *p* < 0.01) (r = −0.410, *p* < 0.01) and between CSI score and both cervical flexo-extension and total rotation (r = −0.363, *p* < 0.01 and r = −0.364, *p* < 0.01 respectively). A moderate positive correlation was also found between SF-12 total score and total cervical flexo-extension and between SF-12 total score with rotation (r = 0.395, *p* < 0.01 and r = 0.431, *p* < 0.01 respectively). A moderate correlation was drawn between all thoracic movements and NDI, CSI, and SF-12 score (*p* < 0.01) (Table 3).

The regression model for NDI is presented in Table 4. The CSI and SF-12 total score were significant covariates for the NDI score (adjusted *R*^2^ = 0.75; *p* < 0.01) (Table 4).

## 4. Discussion

This study has identified a significant reduction in cervical flexo-extension and total rotation AROM and diminished thoracic AROM in an RCRSP group compared to asymptomatic subjects matched by age, gender, and dominant arm. The NDI score was also significantly higher in RCRSP group compared to the asymptomatic group. However, no significant differences were found in cervical lateroflexion, head posture, and cervical retraction and protraction movements. A combination of psychological factors measured by CSI and low quality of life were considered significant predictors of neck disability.

To the best knowledge of the authors, this is the first study to investigate a possible relationship between cervical AROM and RCRSP compared to an asymptomatic group. The importance of cervical physical examination in this population has been recently highlighted in the literature, with a lack of clear cervical spine assessment reported [36]. Shoulder and spine pathology commonly overlap as the neuroanatomic and mechanical connections between these two regions are remarkable [59]. Patients with spine pathology such as nerve injury, disc, facet complex, or paravertebral soft tissues from C3-7 may imitate shoulder pathology but their cause lies in the cervical spine; hence, history and physical examination are key aspects to differentiate between shoulder and neck dysfunction [59].

Mechanical loading of the upper limbs may induce neck pain, increase the mechanical stress on the articular and ligamentous structures of the cervical spine, or trigger a protective muscle spasm [60]. Additionally, recent studies indicate that chronic pain conditions can lead to various changes in the primary motor cortex, such as altered corticospinal excitability, resulting in movement disinhibition and imprecision [61].

This could explain why patients with chronic RCRSP also exhibit altered cervical AROM and NDI compared to an asymptomatic group. Whether cervical AROM restriction is caused by RCRSP or the latter arises as a result of the altered mobility was not in the scope of this study; however, it highlights the importance of including cervical spine assessment in RCRSP populations. Our results did not find a correlation between NDI score and SPADI score. These results differ from those found by Libardoni et al., who found a strong correlation between SPADI total score and NDI in a sample of one hundred and forty volunteers with shoulder pain [62]. The fact that the sample size was fairly larger compared to our study, as it consisted of 140 subjects diagnosed with subacromial impingement syndrome without control group, could explain this discrepancy.

Related to forward head position, our results contrast to Land et al. as we did not find significant differences between both groups [34]. This could be explained by the differences in the assessment method as stated previously in the literature [63]. In our study, a CROM device was utilized in a standardized seated position, whereas Land et al. employed a photograph in standing position.

This study has identified a significant reduction in thoracic AROM in a RCRSP group compared to asymptomatic subjects. Diminished thoracic flexion and extension AROM have been previously reported in the literature [33,34,50]. Choi et al. showed significantly reduced ROM in thoracic spine extension and total ROM compared with the healthy group; however, there was no significant differences in thoracic flexion between groups, measured through X-ray examination [64]. To the best knowledge of the authors, thoracic rotation has not been previously assessed between RCRSP patients and asymptomatic groups. Differences in thoracic flexion, extension, and both rotations can contribute to altered scapular mechanics and shoulder blade movement, increasing stress on the rotator cuff tendons, especially during activities that involve overhead arm movements. This heightened stress can contribute to the development of RCRSP [65].

Chester et al. concluded that psychological factors were consistently associated with shoulder pain patient-rated outcomes measured by the pain self-efficacy questionnaire and patients´ pain beliefs and expectation, highlighting the importance of considering the psychological domain in patients with shoulder pain [66]. These results are in line with our data, as a strong relationship was found between NDI score and CSI and SF-12 score in this population. Thus, patients with RCRSP might exhibit altered NDI score and it can be related to psychological factors, such as lower score in the CSI and low pain self-efficacy. The CSI has demonstrated strong correlation with psychological factors rather than central sensitization processing in patients with shoulder pathology [67]. In addition, psychological factors might influence the perpetuation of pain intensity and disability in chronic shoulder conditions, as suggested by Martinez-Calderon et al. [68]. These findings highlight the importance of assessing and addressing these variables in this population as its implications in patients’ outcome have been reported in previous studies [66].

Our results therefore have implications for clinical practice and future studies. Both cervical and thoracic AROM should be included in RCRSP patients’ physical examination. However, since no relationship was found between SPADI and cervical or thoracic variables, future studies should continue to investigate which spine variable may influence shoulder function and pain.

Some limitations of this study must be acknowledged. First, its cross-sectional design precludes causal inference; thus, future investigations should adopt longitudinal methodologies. Second, the external validity is diminished as our sample only encompasses a particular geographical region. Third, a potential bias related to BMI exists within the current study. In addition, the pain’s quality features were not documented in the present study, warranting their inclusion in future research endeavors. The female group was dominant in the RCRSP group based on the available patients in hospital; this gender discrepancy contrasts with previous studies [34]. No spine X-rays were reported in all RCRSP patients, which could establish a potential cause for spine ROM restriction. Last, since the subjects included in this study presented psychological factors that could influence shoulder pain and disability perpetuation, the results of this article might not be generalizable to the whole spectrum of RCRSP patients.

## 5. Conclusions

A RCRSP group was compared to a group of asymptomatic subjects, matched by age, gender and dominant arm. The RCRSP group had significantly less cervical flexo-extension and total rotation ROM and significantly diminished thoracic AROM. Moderate association was found between cervical flexo-extension and total rotation with NDI, CSI, and SF-12 score. Moderate association was also shown between thoracic AROM with NDI, CSI, and SF-12 score. The variables best explaining neck disability were central sensitization index and quality of life score.

## Figures and Tables

**Table 1:** Demographics characteristics of the sample.

	RCRSP (*n* = 32) Mean ± SD	ASYM (*n* = 32) Mean ± SD	Statistic	*p*-Value
Age (years)	55.47 ± 11.03	56.75 ± 13.41	t = 0.41	0.68
BMI	27.52 ± 5.59	24.12 ± 3.09	t = 3.01	0.04
Onset of pain (months)	39.00 ± 20.04	-		
VAS rest	0.88 ± 1.50	-		
VAS activity	5.97 ± 1.65	-		
Daily life impact	5.72 ± 2.19	-		
CSI	47.84 ± 14.38	19.72 ± 13.26	t = −8.13	<0.01
Total SF-12	29.94 ± 6.63	42.63 ± 3.40	t = 9.63	<0.01
% SF-12	51.09% ± 19.09%	87.44% ± 9.69%	t = 9.61	<0.01
SPADI	58.68 ± 19.38	-		
Gender (female)	25 (78.12%)	24 (75.00%)	*X*^2^ = 0.08	0.77
Dom (right hand)	30 (93.75%)	31 (96.87%)	*X*^2^ = 0.35	0.55

Abbreviations: (ROM) Range of motion; (SD) Standard deviation; (RCRSP) Rotator Cuff Related Shoulder Pain; (BMI) body mass index; (VAS) Visual Analogue Scale; (CSI) Central Sensitization Index; (SF-12) 12-Item Short Form Survey; (SPADI) Shoulder Pain and Disability Index; (Dom) dominance.

**Table 2:** Comparison between groups of physical outcomes.

	RCRSP GROUP Mean ± SD (SEM) *n* = 32	CONTROL GROUP Mean ± SD (SEM) *n* = 32	95% CI	Effect Size	Statistic
Cervical flexo-extension (degrees)	112.47 ± 22.07 (3.49)	128.5 ± 17.85 (2.92)	6.01 to 26.08	d = 0.80	t = 3.20 **
Cervical rotation (degrees)	111.14 ± 22.98 (3.86)	130.23 ± 21.20 (3.69)	8.04 to 30.14	d = 0.86	t = 3.45 **
Cervical lateroflexion (degrees)	68.85 ± 17.58 (2.87)	78.43 ± 20.86 (3.44)	−0.05 to 19.22	d = 0.50	t = 1.99
Neutral position (cm)	18.30 ± 2.28 (0.39)	17.79 ± 1.99 (0.38)	−1.58 to 0.56	d = −0.24	t = −0.95
Protraction (cm)	21.36 ± 2.49 (0.40)	21.54 ± 2.53 (0.45)	−1.07 to 1.44	d = 0.07	t = 0.30
Retraction (cm)	16.08 ± 2.12 (0.36)	15.47 ± 1.79 (0.32)	−1.63 to 0.39	d = −0.30	t = −1.22
Difference (cm)	5.27 ± 2.03 (0.37)	6.07 ± 2.36 (0.40)	−0.37 to 1.98	d = −0.15	t = 1.37
NDI	17.56 ± 7.25 (1.28)	2.47 ± 3.25 (0.52)	−17.90 to −12.29	d = −2.69	t = −10.75 **
Thoracic flexion (cm)	33.02 ± 1.14 (0.19)	34.14 ± 1.01 (0.19)	0.58 to 1.66	d = 1.04	t = 4.16 **
Thoracic extension (cm)	28.63 ± 0.89 (0.14)	27.37 ± 0.89 (0.14)	−1.70 to −0.81	d = −1.40	t = −5.62 **
Thoracic right rotation (degrees)	40.53 ± 10.39 (1.74)	54.45 ± 9.75 (1.80)	8.88 to 18.96	d = 1.38	t = 5.58 **
Thoracic left rotation (degrees)	39.00 ± 11.26 (1.92)	54.10 ± 10.51 (1.92)	9.66 to 20.54	d = 1.39	t = 5.55 **

Abbreviations: (RCRSP) Rotator Cuff Related Shoulder Pain; (SD) Standard Deviation; (SEM) Standard Error of Mean; (CI) Confidence Interval; (NDI) Neck Disability Index. (**) *p*-value < 0.01.

**Table 3:** Correlation Matrix of Pearson Correlation Coefficients.

Pearson’s Correlation	Cx-F/E	Cx-Rot	Cx-LF	Tx-F	Tx-E	Tx-RR	Tx-LR	SPADI	NDI	CSI	SF-12
Cx-F/E	1										
Cx-Rot	0.664 **	1									
Cx-LF	0.541 **	0.666 **	1								
Tx-F	0.369 **	0.367 **	0.056	1							
Tx-E	−0.404 **	−0.484 **	−0.264 *	−0.309 *	1						
Tx-RR	0.455 **	0.504 **	0.224	0.430 **	−0.582 **	1					
Tx-LR	0.555 **	0.575 **	0.352 **	0.439 **	−0.546 **	0.876 **	1				
SPADI	−0.053	−0.042	−0.046	−0.177	−0.038	0.036	−0.121	1			
NDI	−0.407 **	−0.410 **	−0.265 *	−0.403 **	0.642 **	−0.628 **	−0.621 **	0.316	1		
CSI	−0.363 **	−0.364 **	−0.219	−0.386 **	0.511 **	−0.595 **	−0.568 **	0.343	0.818 **	1	
SF-12	0.395 **	0.431 **	0.224	0.387 **	−0.681 **	0.544 **	0.545 **	−0.392 *	−0.812 **	−0.757 **	1

Abbreviations: (Cx-LF) Cervical lateroflexion; (Cx-Rot) Cervical rotation; (Cx-F/E) Cervical flexo-extension; (Tx-F) Thoracic flexion; (Tx-E) Thoracic extension; (Tx-RR) Thoracic right rotation; (Tx-LR) Thoracic left rotation; (SPADI) Shoulder Pain and Disability Index; (NDI) Neck Disability Index; (CSI) Central Sensitization Index; (SF-12) 12-Item Short Form Survey. (*) *p*-value < 0.05 (**) *p*-value < 0.01.

**Table 4:** Multiple backward linear regression analysis for NDI.

			Regression Coefficient (B)	Standarized Coefficient (β)	*p*-Value	VIF	Overall Model		
							Adjusted *R*^2^	*F*	Durbin-Watson
Dependent variable: NDI									
RCRSP and ASYM GROUP	**MODEL**	**Predictor variables**					0.75	94.60	1.60
		CSI	0.23	0.48	<0.01	2.34			
		SF-12	−0–52	−0.45	<0.01	2.34			
		**Excluded variable**							
		Thoracic right rotation		−0.67	0.33	1.20			
		Thoracic left rotation		−0.51	0.47	1.23			

Abbreviations: (RCRSP) Rotator Cuff Related Shoulder Pain; (ASYM) Asymptomatic; (NDI) Neck disability index; (CSI) Central Sensitization Inventory; (SF-12) Quality of life; (VIF) Variance Inflation Factor.

## Data Availability

Data is available by contacting the authors.

## References

[1] Luime J.J., Koes B.W., Hendriksen I.J.M., Burdorf A., Verhagen A.P., Miedema H.S., Verhaar J.A.N. (2004). Prevalence and incidence of shoulder pain in the general population; a systematic review. Scand. J. Rheumatol..

[2] Littlewood C., May S., Walters S. (2013). Epidemiology of rotator cuff tendinopathy: A systematic review. Shoulder Elb..

[3] Lewis J. (2016). Rotator cuff related shoulder pain: Assessment, management and uncertainties. Man. Ther..

[4] Lo C.N., van Griensven H., Lewis J. (2022). Rotator cuff related shoulder pain: An update of potential pathoaetiological factors. New Zeal J. Physiother..

[5] Whittle S., Buchbinder R. (2015). Rotator cuff disease. Ann. Intern. Med..

[6] Diercks R., Bron C., Dorrestijn O., Meskers C., Naber R., De Ruiter T., Willems J., Winters J., Van Der Woude H.J. (2014). Guideline for diagnosis and treatment of subacromial pain syndrome: A multidisciplinary review by the Dutch Orthopaedic Association. Acta Orthop..

[7] Requejo-Salinas N., Lewis J., Michener L.A., La Touche R., Fernández-Matías R., Tercero-Lucas J., Camargo P.R., Bateman M., Struyf F., Roy J.S. (2022). International physical therapists consensus on clinical descriptors for diagnosing rotator cuff related shoulder pain: A Delphi study. Braz. J. Phys. Ther..

[8] Sejersen M.H.J., Frost P., Hansen T.B., Deutch S.R., Svendsen S.W. (2015). Proteomics perspectives in rotator cuff research a systematic review of gene expression and protein composition in human tendinopathy. PLoS ONE.

[9] Russell R.D., Knight J.R., Mulligan E., Khazzam M.S. (2014). Structural integrity after rotator cuff repair does not correlate with patient function and pain: A meta-analysis. J. Bone Jt. Surg. Am..

[10] Minagawa H., Yamamoto N., Abe H., Fukuda M., Seki N., Kikuchi K., Kijima H., Itoi E. (2013). Prevalence of symptomatic and asymptomatic rotator cuff tears in the general population: From mass-screening in one village. J. Orthop..

[11] Hegedus E.J., Cook C., Brennan M., Wyland D., Garrison J.C., Driesner D. (2010). Vascularity and tendon pathology in the rotator cuff: A review of literature and implications for rehabilitation and surgery. Br. J. Sports Med..

[12] May S., Chance-Larsen K., Littlewood C., Lomas D., Saad M. (2010). Reliability of physical examination tests used in the assessment of patients with shoulder problems: A systematic review. Physiotherapy.

[13] Tempelhof S., Rupp S., Seil R. (1999). Age-related prevalence of rotator cuff tears in asymptomatic shoulders. J. Shoulder Elb. Surg..

[14] Hegedus E.J., Goode A., Campbell S., Morin A., Tamaddoni M., Moorman C.T., Cook C. (2008). Physical examination tests of the shoulder: A systematic review with meta-analysis of individual tests. Br. J. Sports Med..

[15] Hegedus E.J., Goode A.P., Cook C.E., Michener L., Myer C.A., Myer D.M., Wright A.A. (2012). Which physical examination tests provide clinicians with the most value when examining the shoulder? Update of a systematic review with meta-analysis of individual tests. Br. J. Sports Med..

[16] Clark J.M., Harryman D.T. (1992). Tendons, ligaments, and capsule of the rotator cuff. Gross and microscopic anatomy. J. Bone Jt. Surg. Ser. A.

[17] Clark J., Sidles J.A., Matsen F.A. (1990). The relationship of the glenohumeral joint capsule to the rotator cuff. Clin. Orthop. Relat. Res..

[18] Boettcher C.E., Ginn K.A., Cathers I. (2009). The ‘empty can’ and ‘full can’ tests do not selectively activate supraspinatus. J. Sci. Med. Sport..

[19] Lewis J.S. (2009). Rotator cuff tendinopathy/subacromial impingement syndrome: Is it time for a new method of assessment?. Br. J Sports Med..

[20] McLean S.M., Moffett J.K., Sharp D.M., Gardiner E. (2011). An investigation to determine the association between neck pain and upper limb disability for patients with non-specific neck pain: A secondary analysis. Man. Ther..

[21] Hwang S., Mun M.-H. (2013). Relationship of neck disability index, shoulder pain and disability index, and visual analogue scale in individuals with chronic neck pain. Phys. Ther. Rehabil. Sci..

[22] Vogt M.T., Simonsick E.M., Harris T.B., Nevitt M.C., Kang J.D., Rubin S.M., Kritchevsky S.B., Newman A.B. (2003). Neck and shoulder pain in 70- to 79-year-old men and women: Findings from the health, aging and body composition study. Spine J..

[23] Zhang A.L., Theologis A.A., Tay B., Feeley B.T. (2015). The association between cervical spine pathology and rotator cuff dysfunction. J. Spinal Disord. Tech..

[24] Littlewood C., Bateman M., Brown K., Bury J., Mawson S., May S., Walters S.J. (2016). A self-managed single exercise programme versus usual physiotherapy treatment for rotator cuff tendinopathy: A randomised controlled trial (the SELF study). Clin. Rehabil..

[25] Crawshaw D.P., Helliwell P.S., Hensor E.M.A., Hay E.M., Aldous S.J., Conaghan P.G. (2010). Exercise therapy after corticosteroid injection for moderate to severe shoulder pain: Large pragmatic randomised trial. BMJ.

[26] Ekeberg O.M., Bautz-Holter E., Tveitå E.K., Juel N.G., Kvalheim S., Brox J.I. (2009). Subacromial ultrasound guided or systemic steroid injection for rotator cuff disease: Randomised double blind study. BMJ.

[27] Date E., Gray L. (1996). Electrodiagnostic evidence for cervical radiculopathy and suprascapular neuropathy in shoulder pain. Electromyogr. Clin. Neurophysiol..

[28] Gumina S., Carbone S., Arceri V., Rita A., Vestri A.R., Postacchini F. (2009). The relationship between chronic type III acromioclavicular joint dislocation and cervical spine pain. BMC Musculoskelet. Disord..

[29] Crawford H.J., Jull G.A. (1993). The influence of thoracic posture and movement on range of arm elevation. Physiother. Theory Pract..

[30] Kebaetse M., McClure P., Pratt N.A. (1999). Thoracic position effect on shoulder range of motion, strength, and three-dimensional scapular kinematics. Arch. Phys. Med. Rehabil..

[31] Stewart S.G., Jull G.A., Ng J.K.F., Willems J.M. (1995). An initial analysis of thoracic spine movement during unilateral arm elevation. J. Man. Manip. Ther..

[32] Barrett E., O’Keeffe M., O’Sullivan K., Lewis J., McCreesh K. (2016). Is thoracic spine posture associated with shoulder pain, range of motion and function? A systematic review. Man. Ther..

[33] Hunter D.J., Rivett D.A., McKeirnan S., Smith L., Snodgrass S.J. (2020). Relationship between shoulder impingement syndrome and thoracic posture. Phys. Ther..

[34] Manoso-Hernando D., Bailón-Cerezo J., Angulo-Díaz-Parreño S., Reina-Varona Á., Elizagaray-García I., Gil-Martínez A. (2024). Shoulder mobility and strength impairments in patients with rotator cuff related shoulder pain: A systematic review and meta analysis. PeerJ.

[35] Kulkarni R., Gibson J., Brownson P., Thomas M., Rangan A., Carr A.J., Rees J.L. (2015). Subacromial shoulder pain. Shoulder Elb..

[36] Walker T., Salt E., Lynch G., Littlewood C. (2019). Screening of the cervical spine in subacromial shoulder pain: A systematic review. Shoulder Elb..

[37] von Elm E., Altman D.G., Egger M., Pocock S.J., Gøtzsche P.C., Vandenbroucke J.P. (2008). The strengthening the reporting of observational studies in epidemiology (STROBE) statement: Guidelines for reporting observational studies. J. Clin. Epidemiol..

[38] Roach K.E., Budiman-Mak E., Songsiridej N., Lertratanakul Y. (1991). Development of a shoulder pain and disability index. Arthritis Care Res..

[39] Vernon H., Mior S. (1991). The Neck Disability Index: A study of reliability and validity. J. Manip. Physiol. Ther..

[40] Ware J.E., Kosinski M., Keller S.D. (1996). A 12-Item Short-Form Health Survey: Construction of scales and preliminary tests of reliability and validity. Med. Care.

[41] Neblett R. (2018). The central sensitization inventory: A user’s manual. J. Appl. Biobehav. Res..

[42] Jensen M.P., Karoly P., Braver S. (1986). The measurement of clinical pain intensity: A comparison of six methods. Pain.

[43] Blanpied P.R., Gross A.R., Elliott J.M., Devaney L.L., Clewley D., Walton D.M., Sparks C., Robertson E.K. (2017). Neck Pain: Revision 2017. J. Orthop. Sports Phys. Ther..

[44] Fletcher J.P., Bandy W.D. (2008). Intrarater reliability of CROM measurement of cervical spine active range of motion in persons with and without neck pain. J. Orthop. Sports Phys. Ther..

[45] Hole D.E., Cook J.M., Bolton J.E. (1995). Reliability and concurrent validity of two instruments for measuring cervical range of motion: Effects of age and gender. Man. Ther..

[46] Youdas J.W., Carey J.R., Garrett T.R. (1991). Reliability of measurements of cervical spine range of motion—Comparison of three methods. Phys. Ther..

[47] Reese N.B., Bandy W.D. (2016). Joint Range of Motion and Muscle Length Testing.

[48] Elizagaray-García I., Obispo-Villamayor Á., Prats-Martínez C., Prieto-Hernández G., Carvalho G.F. (2024). Measurement proprieties of the CROM instrument for assessing head posture, neck retraction and protraction. Musculoskelet. Sci. Pract..

[49] Youdas J.W., Garrett T.R., Suman V.J., Bogard C.L., Hallman H.O., Carey J.R. (1992). Normal range of motion of the cervical spine: An initial goniometric study. Phys. Ther..

[50] Theisen C., Van Wagensveld A., Timmesfeld N., Efe T., Heyse T.J., Fuchs-Winkelmann S., Schofer M.D. (2010). Co-occurrence of outlet impingement syndrome of the shoulder and restricted range of motion in the thoracic spine—A prospective study with ultrasound-based motion analysis. BMC Musculoskelet. Disord..

[51] do Valle M.B., Detogni Schmit E.F., Sedrez J.A., Candotti C.T. (2019). Assessment of thoracic and lumbar spine range of motion: Systematic review with meta-analysis. J. Phys. Educ..

[52] Johnson K.D., Kim K.M., Yu B.K., Saliba S.A., Grindstaff T.L. (2012). Reliability of thoracic spine rotation range-of-motion measurements in healthy adults. J. Athl. Train..

[53] Koo T.K., Li M.Y. (2016). A Guideline of selecting and reporting intraclass correlation coefficients for reliability research. J. Chiropr. Med..

[54] Faul F., Erdfelder E., Lang A.G., Buchner A. (2007). G*Power 3: A flexible statistical power analysis program for the social, behavioral, and biomedical sciences. Behav. Res. Methods.

[55] Altman D.G. (1990). Practical Statistics for Medical Research.

[56] Sani F., Todman J.B. (2006). Experimental Design and Statistics for Psychology: A First Course.

[57] Valiente L.P., Tejedor I.H. (2010). Bioestadisticas sin Dificultades Matematicas.

[58] Kiernan D. (2014). Chapter 7: Correlation and simple linear regression. Textbooks OS.

[59] Katsuura Y., Bruce J., Taylor S., Gullota L., Kim H.J. (2020). Overlapping, Masquerading, and causative cervical spine and shoulder pathology: A systematic review. Glob. Spine J..

[60] Gorski J.M., Schwartz L.H. (2003). Shoulder impingement presenting as neck pain. J. Bone Jt. Surg. Am..

[61] Moseley G.L., Flor H. (2012). Targeting cortical representations in the treatment of chronic pain: A review. Neurorehabilit. Neural Repair.

[62] Libardoni T.d.C., Armijo-Olivo S., Bevilaqua-Grossi D., de Oliveira A.S. (2020). Relationship between intensity of neck pain and disability and shoulder pain and disability in individuals with subacromial impingement symptoms: A Cross-Sectional Study. J. Manipulative Physiol. Ther..

[63] Shaghayeghfard B., Ahmadi A., Maroufi N., Sarrafzadeh J. (2016). Evaluation of forward head posture in sitting and standing positions. Eur. Spine J..

[64] Choi M., Chung J. (2023). Biomechanical and functional analysis of the shoulder complex and thoracic spine in patients with subacromial impingement syndrome: A case control study. Medicine.

[65] Howe L., Read P. (2015). Thoracic spine function: Assessment and self-management. Prof. J. Strength Cond..

[66] Chester R., Jerosch-Herold C., Lewis J., Shepstone L. (2018). Psychological factors are associated with the outcome of physiotherapy for people with shoulder pain: A multicentre longitudinal cohort study. Br. J. Sports Med..

[67] Coronado R.A., George S.Z. (2018). The central sensitization inventory and pain Sensitivity questionnaire: An exploration of construct validity and associations with widespread pain sensitivity among individuals with shoulder pain. Musculoskelet. Sci. Pract..

[68] Martinez-Calderon J., Meeus M., Struyf F., Miguel Morales-Asencio J., Gijon-Nogueron G., Luque-Suarez A. (2018). The role of psychological factors in the perpetuation of pain intensity and disability in people with chronic shoulder pain: A systematic review. BMJ Open.

